# Determination of Chemical Composition and Antioxidant Capacity of Fruit Tree Resins Belonging to the Prunus Species and Determination of Their In Vivo Diuretic Activities

**DOI:** 10.3390/molecules31173004

**Published:** 2026-08-27

**Authors:** Sadık Küçükgünay, Demirel Ergün, Fatma Ergün

**Affiliations:** 1 Department of Medical Pharmacology, Faculty of Medicine, Kırsehir Ahi Evran University, 40100 Kırşehir, Türkiye; 2 Experimental Animals Unit, Faculty of Medicine, Kırsehir Ahi Evran University, 40100 Kırşehir, Türkiye; demirel.ergun@ahievran.edu.tr; 3 Department of Nutrition and Dietetics, Faculty of Health Sciences, Kırşehir Ahi Evran University, 40100 Kırşehir, Türkiye; fatma.ergun@ahievran.edu.tr

**Keywords:** tree resin, antioxidant effect, diuretic effect, natriuretic activity, saluretic index

## Abstract

This study aimed to evaluate the phenolic content, mineral profile, antioxidant capacity, and in vivo diuretic activity of resins secreted by *Rosaceae* family trees grown in the same environment. Total phenolic and flavonoid contents were determined using Folin–Ciocalteu and aluminum nitrate methods, respectively. Antioxidant capacity was evaluated via iron-reducing power, copper-reducing capacity, and DPPH assays. Resin chemical composition, phenolic structures, and mineral content were analyzed using GC/MS, HPLC, and ICP-MS. For the in vivo diuretic activity, Wistar albino rats were divided into control (0.9% NaCl), furosemide (10 mg/kg), and resin extract (100 mg/kg) groups. Urine samples were collected at 5 and 24 h to analyze diuretic activity, creatinine, urea, uric acid, sodium, potassium, and chloride levels. Plant resins demonstrated high phenolic and flavonoid contents, strong antioxidant properties, and rich essential mineral profiles with valuable bioactive compounds. Furthermore, the resins exhibited potential diuretic activity parallel to that of furosemide. At 24 h, urine activity was high in *Prunus spinosa* and *Prunus dulcis*, while *Prunus domestica*, *Prunus cerasifera*, *Prunus armeniaca*, and *Prunus persica* showed moderate activity. *Prunus* plant resins possess significant potential as bioactive components, natural antioxidants, or preservatives in food formulations. Additionally, their demonstrated diuretic effect highlights their promising therapeutic potential in fluid balance management.

## 1. Introduction

Diuretics increase urine production in the body, providing a positive risk–benefit relationship and playing an important role in facilitating the excretion of water and electrolytes from the body [1]. Therefore, they are used in the management of some important conditions, especially some edematous conditions, as well as hypertension, congestive heart failure, and kidney disorders [2]. When used, they have side effects on the organism, as with other pharmaceutical agents. The most common side effects include hypovolemia, body lipid profile irregularities, and body electrolyte and acid–base balance disorders [3]. Their effects on the body lipid profile can cause a decrease in HDL-cholesterol levels and an increase in triglyceride, total cholesterol, and LDL-cholesterol levels [4]. Athletes who use these drugs have also reported experiencing hypovolemia, fatigue, and muscle cramps [5]. As with other medications, the potential side effects of these drugs have led to a growing search for natural alternatives and an increase in research in this area. In addition, the literature reports that plant-derived antioxidant compounds contribute to cellular defense by neutralizing free radicals, and also exhibit diuretic effects by modulating renal blood flow or regulating tubular reabsorption [6,7]. In this context, many plants and their by-products rich in secondary metabolites have long been used as diuretic agents [8]. In addition, studies have reported that plant-based drugs show higher efficacy and preferability compared to their synthetic counterparts [9]. Furthermore, the fact that plants can be used as potential sources with diuretic properties is supported by numerous studies [10,11].

**Figure 1:**
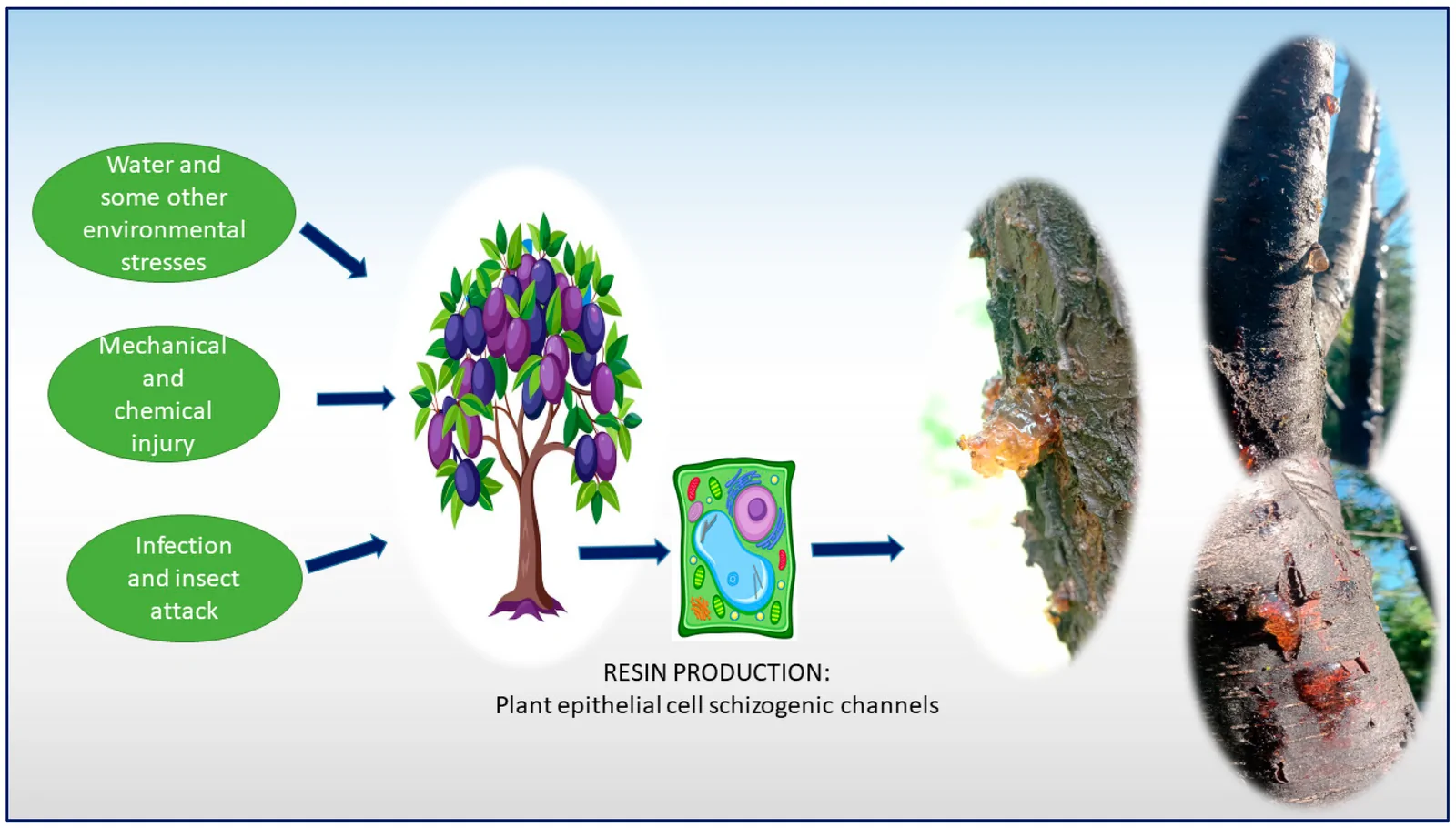
Resin biosynthesis and secretion mechanism in plants.

Resins, also known as gums, are crucial plant structures among the large-molecule exogenous and endogenous secretions that plants release for self-protection. These structures are secreted from the epithelial cells of plants to adapt to environmental stresses, trauma, and pathogens and are stored and secreted from schizogenic channels and special vesicles within the cells (Figure 1) [12,13]. Many plants of the *Prunus* genus, belonging to the *Rosaceae* family, secrete abundant resins as exogenous secretions for protection [14]. These secretions are in the form of tear-like, amorphous lumps. Initially soft and white to light yellow in color, these structures darken and harden over time. These structures, called gums or resins, are non-toxic and have a sweet and slightly bitter taste. They are known to have functions such as strengthening the immune system and lowering blood sugar and lipids [15,16].

The resin secreted by the apricot tree (*P. armeniaca*) has long been used in traditional folk medicine for its various therapeutic properties as an antidote, cough suppressant, and fever reducer [17]. It is also believed to treat constipation and anemia, stop bleeding, treat some tumors, and increase fertility [18]. The gum of *P. domestica* has traditionally been used as a tonic, laxative, and anthelmintic [18,19]. Iranian gum, the natural form of the resin of a wild almond species (*Prunus scoparia*), is used in Iran and some other countries as a herbal remedy for swollen joints (as a poultice), antiparasitic, toothache reliever, appetite stimulant, cough suppressant, hair conditioner, and skin brightener [20]. In Central Asia, and especially in ancient China, peach (*P. persica*) resin was regarded as part of Chinese medicine and believed to be able to cure diseases [15]. In addition to traditional uses of plant resins, studies have generally concluded that resins can be used in the food industry to improve viscosity, texture, and stability in formulations, as well as to form films, coatings, and gels [21,22]. The stabilization ability of almond (*P. dulcis*) resin in milk–orange juice and milk–sour cherry juice mixtures has also been reported [23]. Furthermore, numerous studies have confirmed that peach (*P. persica*) resin is a key functional element in the treatment of many diseases and has the potential to prevent hyperglycemia and hyperlipidemia and treat urinary difficulties [15,16]. Furthermore, it has been reported that peach (*P. persica*) resin exhibits excellent emulsification, antioxidant, antibacterial, antidiabetic, and hypolipidemic properties, and that it could be a promising edible raw material in the field of bioplastic research and development, given the alarming level of plastic pollution on a global scale [24]. Similarly, it has been determined that resins obtained from the trunks of *P. armeniaca* trees can be used in food components as fat substitutes, emulsifiers, stabilizers, edible coatings, and thickeners [25,26].

This research, one of the pioneering studies investigating the diuretic potential of plant exudates (secretions), focuses on specific fruit tree resins of the *Prunus* species, which have a high potential for diuretic activity due to their rich antioxidant profile and possible mineral content. The objective of this study is to determine the in vitro antioxidant capacities and mineral compositions of resins from apricot (*P. armeniaca*—PAR), green plum (*P. cerasifera* Ehr.—PCR), blackthorn (*P. spinosa*—PSR), black plum (*P. domestica*—PDoR), almond (*P. dulcis*—PDuR), and peach (*P. persica*—PPR) trees, and to evaluate their in vivo diuretic activity in an animal model.

## 2. Results and Discussion

In the study, the total phenolic and flavonoid content, CUPRAC, FRAP activities, and IC_50_ values of the plant resins were determined (Table 1).

In the study, the differences in the total phenolic content of the plant resins were significant [F(5,12) = 164.816, *p* = 0.001] (Table 1). The highest value was found in PSR and the lowest value in PPR. In similar studies, the total phenolic content was reported as 1.14 mg–1.17 mg GAE/g in Malatya apricot (*Prunus armeniaca*) resin, 64 ± 2.33 mg/g in *P. cerasoides* resin, and 58.33 ± 2.4 mg GAE/g in *Prunus amygdalus* resin [27,28,29]. Although the values we obtained belonged to different types of plant resins, they are higher than those reported in the literature. It is believed that several dynamic factors contribute to these differences, including genetic structure (species and variety), environmental stressors (abiotic and biotic stresses), harvest time, plant organ, and post-harvest processing methods. This is because plants produce secondary metabolites such as phenolic compounds and flavonoids to protect themselves from oxidative damage caused by free radicals; this production mechanism constantly changes depending on external factors.

In the study, the differences in the total flavonoid amounts of plant resins were found to be significant [F(5,12) = 23.687, *p* = 0.001] (Table 1). The highest value was found to be 65.00 ± 3.00 mg QE/g DE in PDuR, and the lowest value was 32.00 ± 2.64 mg QE/g DE in PDoR. In a similar study, the total flavonoid amount of almond tree gums was reported to be 11.43 ± 1.4 mg QE/g [28]. The total flavonoid amount of 65.47 ± 2.51 mgQE/g DE in almond tree gums that we found in our study is higher than that found by Bouaziz et al. (2017) [28].

Significant differences were found in the FRAP activities of plant resins [F(6,14) = 272.047, *p* = 0.001] (Table 1). In our study, the value we determined as 3.61 ± 0.01 mmol TE/g extract in the PSR resin extract is higher than the BHT value we used as a standard. High FRAP activity was also determined in other extracts except PPR extract. The reduction activity is in the order of PSR > BHT > PDuR > PCR > PDoR > PAR > PPR. Similarly, studies conducted with different plant resins have reported Fe II values of 0.09 and 0.06 mg AAE/g for guar gum, 0.12 and 0.09 mg AAE/g for locust bean gum, and 916.5 ± 28.7 µmol Fe II/g for pistachio (*Pistacia vera* L.) resin [30,31]. The values we found are higher than these. It is thought that the plant species has an effect on this difference.

In the study, the copper (II) ion reduction-based antioxidant capacities (CUPRAC) of plant resins were determined, and the differences between the groups were found to be significant [F(6,14) = 520.610, *p* = 0.001] (Table 1). The highest value was found in PDuR and the lowest value in PPR. The DPPH radical scavenging inhibition of plant resins exhibited a clear concentration-dependent increase across the tested concentration range (25–150 µg/mL), demonstrating high linearity as evaluated by linear regression analysis ($R^{2}>0.98$). According to the DPPH method, the antioxidant activity was determined to be in the order of PDuR > PSR > PCR > PDoR > PAR > BHT > PPR. In the study, the inhibition concentration (IC_50_) values for plant gums were calculated, and the differences between the groups were found to be significant [F(6,14) = 351.199, *p* = 0.001] (Table 1). In this study, the IC_50_ values of BHT, used as a standard, were determined as 101.66 ± 5.83 mg/mL, while the IC_50_ values of all resins except PPR were calculated to be lower than that of BHT. The IC_50_ value is inversely proportional to the radical scavenging activity. Therefore, it was determined that the radical scavenging capacities of the PDuR, PSR, PCR, PDoR, and PAR resins used in the study were higher than those of BHT, which was used as a standard. Across all evaluated assays, a clear parallel was observed between total bioactive contents and antioxidant capacities; extracts possessing high TPC and TFC values (notably PDuR and PSR) consistently demonstrated higher reducing potentials (FRAP and CUPRAC) and superior radical scavenging capacity (lower ${\mathrm{IC}}_{50}$ values), supporting the direct contribution of the phenolic matrix to the overall antioxidant performance. Notably, all assay parameters (incubation period, solvent composition, and spectrophotometric readings) were maintained strictly identical for both BHT and the resin extracts to guarantee precise comparison. This superior radical scavenging activity observed in specific resins compared to the commercial synthetic antioxidant BHT can be attributed to the complex synergistic effects of diverse bioactive compounds within the resin matrices. Natural plant gums and resins often contain a rich mixture of multi-hydroxylated phenolic acids, flavonoids, and polymeric compounds, which can donate hydrogen or electrons more efficiently than a single synthetic molecule like BHT [29]. This makes these resins strong radical scavengers, hydrogen donors, reactive oxygen species quenchers, and metal ion chelators.

**Figure 2:**
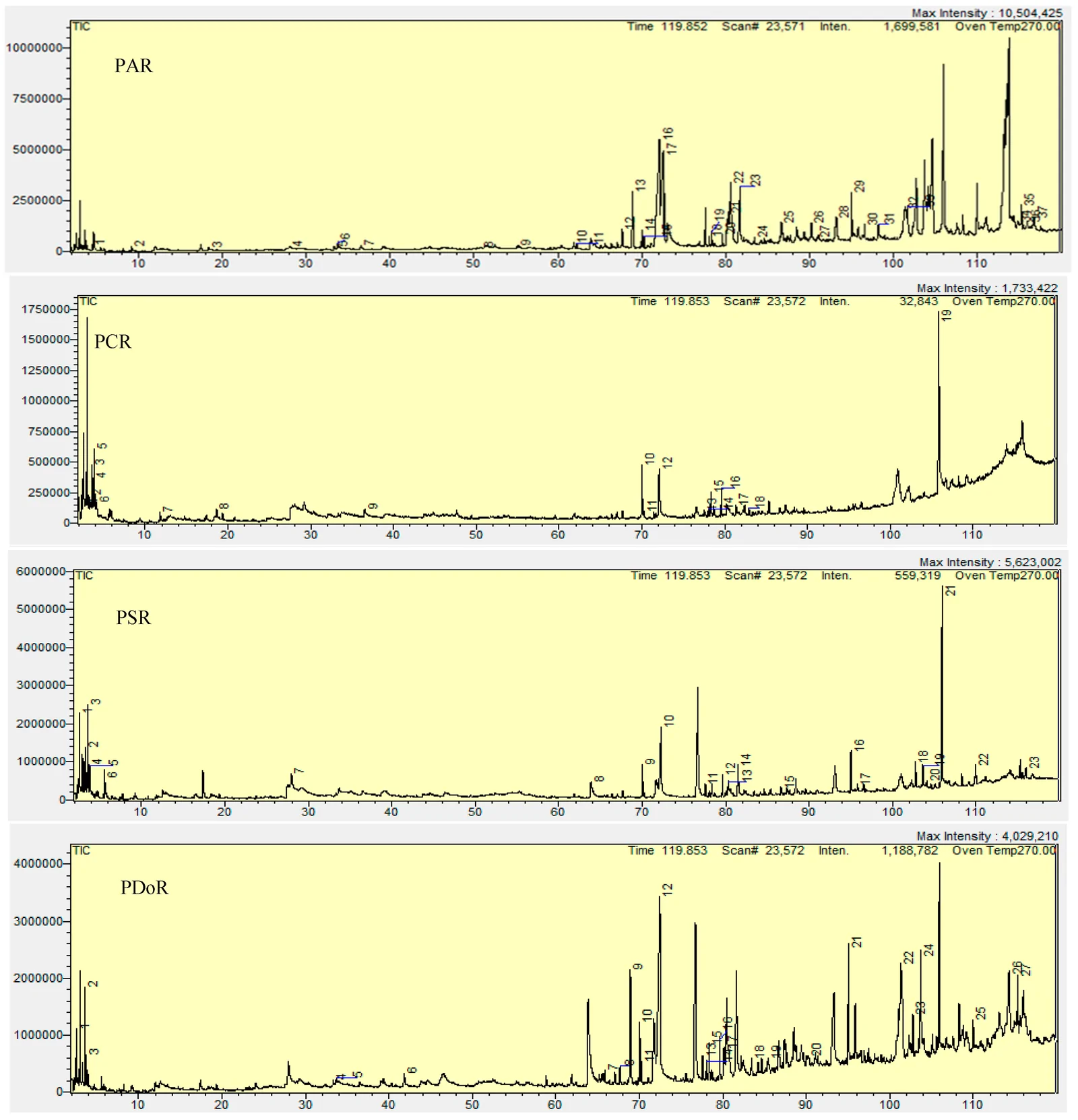

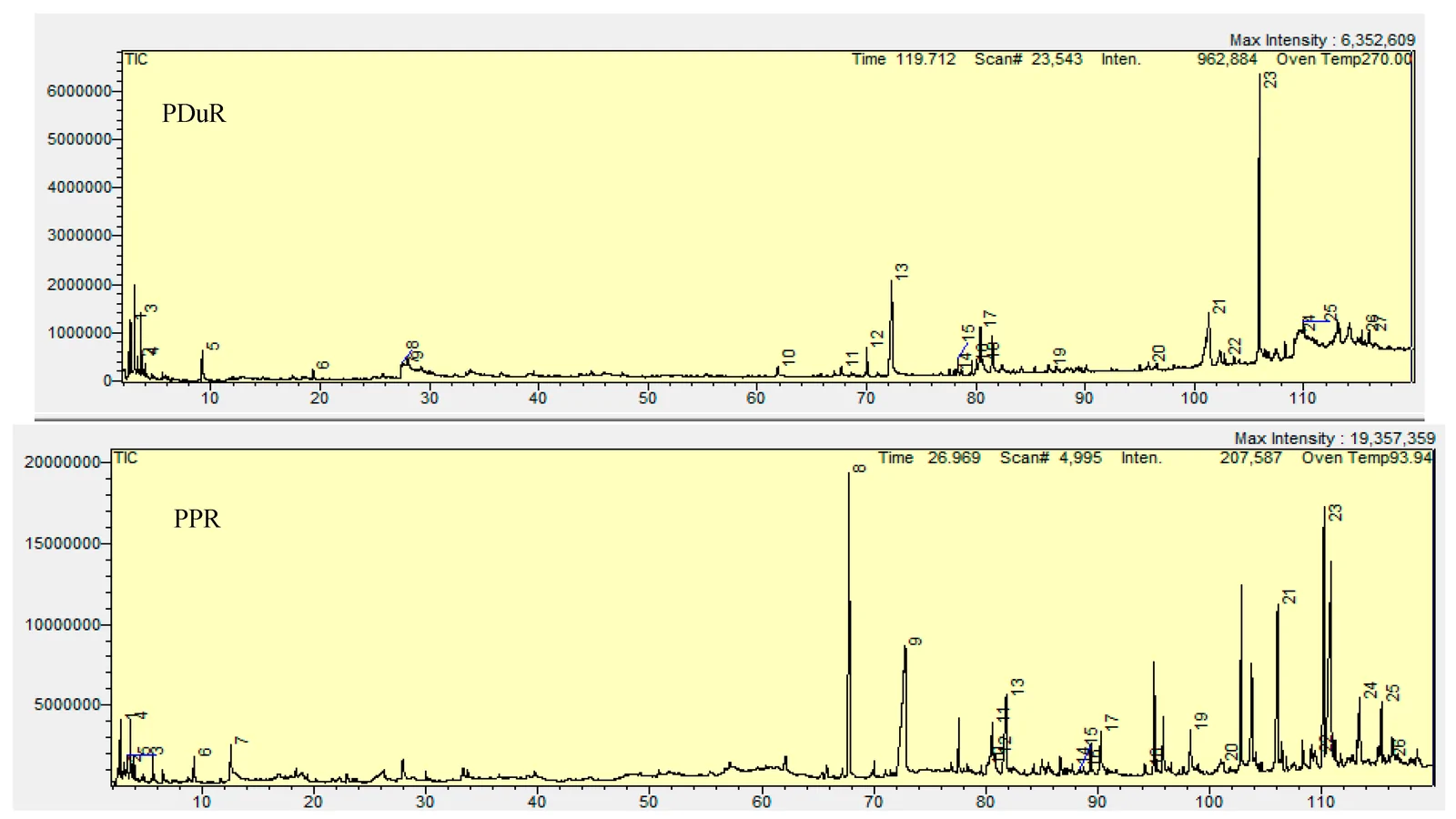
Chromatograms obtained from resin samples using the GC-MS method (PAR: *P. armeniaca* resin, PCR: *P. cerasifera Ehr.* resin, PSR: *P. spinosa* resin, PDoR: *P. domestica* resin, PDuR: *P. dulcis* resin, PPR: *P. persica* resin).

Examination of the GC-MS analysis results revealed that the plant resin samples PAR, PDoR, PSR, PCR, PDuR, and PPR exhibited both common dominant components and significant species-specific variations in their chemical composition (Figure 2, Table 2). The most striking finding in the overall profile of the extracts was the predominance of fatty acids and amide derivatives. In particular, the 9-octadecenamide (Z) compound stood out as the major component detected in the highest amounts in the PCR (32.38%), PSR (24.02%), and PDuR (30.97%) extracts. Similarly, n-Hexadecanoic acid (Palmitic acid), a saturated fatty acid, was detected in high amounts in all species studied; The highest accumulation of this component was observed in the PDoR extract (13.45%), followed by PPR (8.00%), PAR (11.65%), PDuR (10.44%), PSR (7.89%), and PCR (8.33%), respectively.

GC-MS analysis revealed that compounds such as 2H-1-benzopyran-2-one (a coumarin derivative), 7-hydroxy-6-methoxy-(scopoletin), naringenin, 9-octadecenamide, (Z)-(oleamide), fatty acids, and β-sitosterol directly contribute to the diuretic effect in rats. These substances increase blood flow to the kidneys and prevent the reabsorption of sodium and water in the renal tubules. As a result, urine volume increases and electrolyte excretion accelerates in rats. In short, these identified bioactive components stimulate the kidneys, creating a potent diuretic effect.

**Figure 3:**
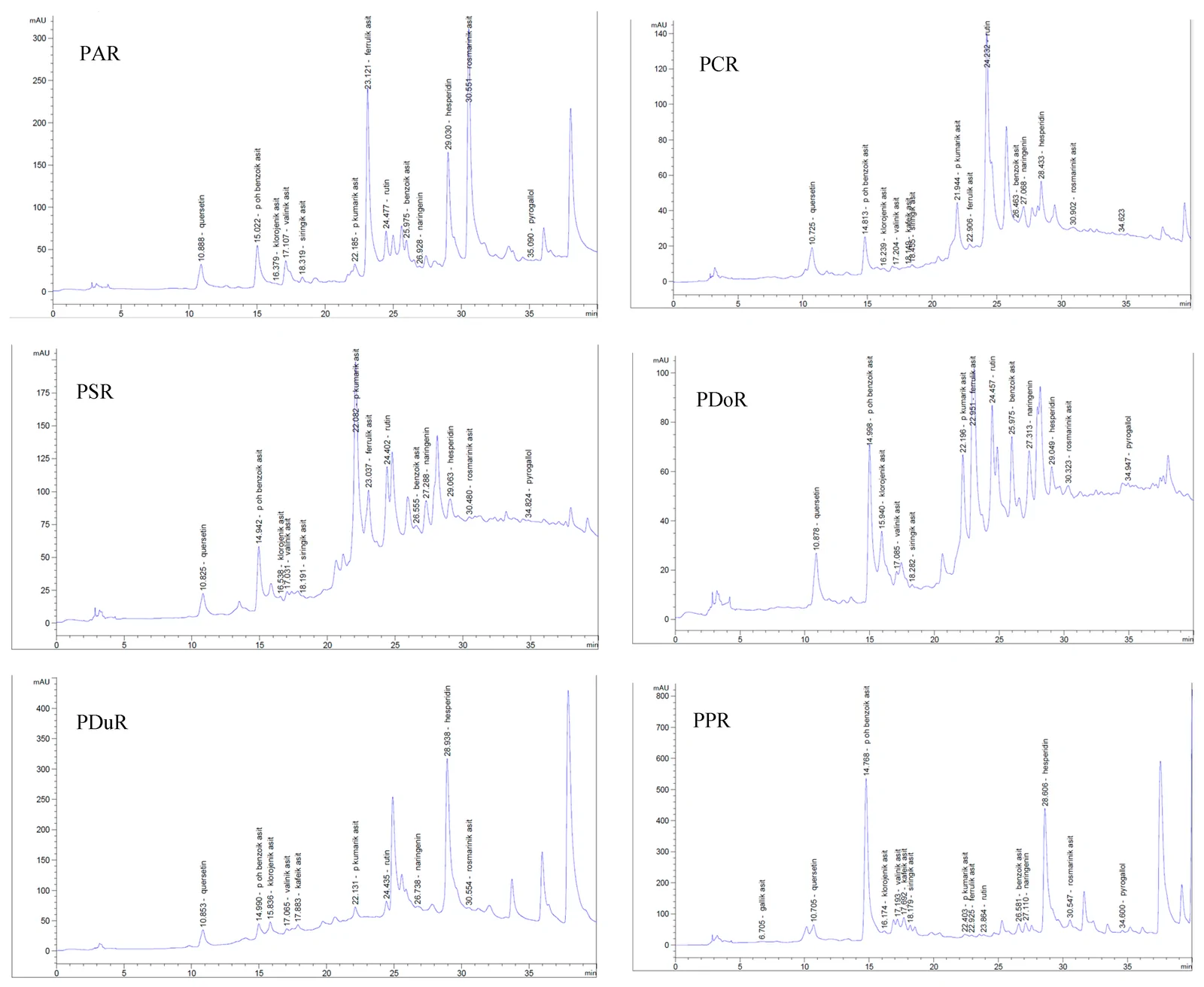
Chromatograms obtained from resin samples using the HPLC method (PAR: *P. armeniaca* resin, PCR: *P. cerasifera Ehr.* resin, PSR: *P. spinosa* resin, PDoR: *P. domestica* resin, PDuR: *P. dulcis* resin, PPR: *P. persica* resin).

HPLC analysis results show that the resin samples (PAR, PDoR, PSR, PCR, PDuR, and PPR) exhibit a rich distribution in terms of phenolic compound profile and amounts (mg/kg dry weight), showing species-specific variations (Figure 3; Supplementary Table S1). When the overall profile of the extracts is examined, hesperidin, a glycosidic flavanone, stands out as the major component detected in the highest concentration in almost all species. Hesperidin content reaches high levels in PPR (35,289.8 mg/kg) and PDuR (30,177.1 mg/kg) extracts, followed by PAR (10,166.60 mg/kg), PSR (2171.79 mg/kg), PDoR (1729.53 mg/kg), and PCR (769.48 mg/kg). The presence of such high concentrations of hesperidin, known in the literature for its strong anti-inflammatory, antioxidant, and vascular protective effects, in *Prunus* gums indicates the high-value biomedical or food additive potential of these byproducts. When the phenolic acid composition of the extracts is examined, the predominance of p-coumaric acid, a derivative of hydroxycinnamic acid, stands out. The amount of p-coumaric acid peaks particularly in the PCR (4707.44 mg/kg) and PDoR (4686.48 mg/kg) extracts, while it is detected in similar and high concentrations in the PSR (2746.90 mg/kg) and PDuR (2741.02 mg/kg) samples. Although hesperidin represents the predominant flavanone across these resins, the overall biological potential—including antioxidant and potential biomedical efficacy—is governed by the synergistic interplay of the total phenolic matrix rather than a single constituent in isolation. For instance, extracts such as PDuR, which combine exceptionally high hesperidin levels with rich total flavonoid and phenolic contents, exhibited the highest radical scavenging capacity (lowest IC_50_) and reducing power (FRAP and CUPRAC). Conversely, in samples where overall phenolic diversity is lower, the individual contribution of hesperidin is complemented by hydroxycinnamic derivatives such as p-coumaric acid. This multi-component synergistic mechanism demonstrates that while hesperidin serves as a primary marker compound, the collective phenolic profile is responsible for the potent biological potential of *Prunus* resins.

In this study, ICP-MS analyses were performed to determine the amounts of macro elements (Ca, K, Mg, Na, Fe, and P), micro elements (Cu and Zn), and trace elements (Cr and Mn) present in plant resins. It was determined that the highest amounts of Na, P, Ca, and Cr were found in PAR, the highest amounts of Mg and K in PCR, the highest amounts of Fe and Cu in PSR, the highest amount of Zn in PDoR, and the highest amount of Mn in PPS (Table 3).

A study investigating macro- and micronutrients in various natural resin samples from Pakistan reported that the minerals found in *P. armeniaca* and *P. persica* resins were ranked as K > Na > Mg > P > Ca > S > Fe and in *P. domestica* resin as K > P > Mg > Ca > Na > Fe > S [32]. Furthermore, in another study, the mineral content of *P. armeniaca* resin was reported as Sodium (Na) 112.39 ± 1.12 ppm, Magnesium (Mg) 3556.6 ± 1.97 ppm, Phosphorus (P) 424.00 ± 1.32 ppm, Potassium (K) 245.80 ± 0.26 ppm, Calcium (Ca) 11,043.41 ± 1.62 ppm, Chromium (Cr) 0.61 ± 0.00 ppm, Manganese (Mn) 30.71 ± 0.00 ppm, Iron (Fe) 963.00 ± 0.21 ppm, Copper (Cu) 0.25 ± 0.00 ppm, and Zinc (Zn) 198.92 ± 0.06 ppm [33]. The values we found in this study are similar to those found by Fathi et al., 2016 [33]. Furthermore, the pattern in the mineral content of the plant resins we found in this study is similar to the pattern reported by Jamila et al., 2020 [32]. It should be noted that while trace elements such as Fe, Cu, Zn, and Mn are recognized in the literature as essential cofactors in biological redox processes and enzyme activation, no direct statistical correlation was performed between the mineral profile and the observed bioactivities in the present study. Therefore, the mineral composition is provided as a fundamental chemical characterization of the resins rather than a confirmed primary cause of their antioxidant or diuretic capacities.

Urine volumes accumulated during the application period were measured in all experimental groups, and significant differences were found between the groups at 5 h [F(7,32) = 37.449, *p* = 0.001] and 24 h [F(7,32) = 9.917, *p* = 0.001] (Table 4).

The highest urine output was observed in animals treated with furosemide at 5 h and in animals treated with blackthorn (PSR) plant resin at 24 h. Diuretic effect and diuretic activity values for the treatment groups were calculated at 5 and 24 h and are expressed in detail in Table 4. In the study, where the control group was considered as 1 in terms of diuretic effect, the highest diuretic effect was calculated in the group treated with furosemide at 5 h [F(7,32) = 25.197, *p* = 0.001] (2.06 ± 0.11) and in the group treated with blackthorn (PSR) plant resin at 24 h [F(7,32) = 24.621, *p* = 0.001] (3.64 ± 0.55). Similarly, in the study where furosemide was considered as 1 in terms of diuretic activity, the highest diuretic activity value was measured as 0.76 ± 0.14 and 1.17 ± 0.09 in the group treated with blackthorn (PSR) plant resin at the end of 5 h [F(7,32) = 10.268, *p* = 0.001] and 24 h [F(7,32) = 11.187, *p* = 0.001], respectively. According to the literature, numerical results regarding diuretic activity are categorized as follows: diuretic activity less than 0.50 is considered ineffective, between 0.50 and 0.69 is considered low, between 0.70 and 0.89 is considered moderate, and equal to or greater than 0.90 is considered high [34,35]. After 24 h, it was determined that the resin extracts of blackthorn (*P. spinosa*) (PSR) (1.17 ± 0.09) and almond (*P. dulcis*) (PDuR) (1.00 ± 0.14) had high diuretic activity, while the resin extracts of black plum (*P. domestica*) (PDoR) (0.79 ± 0.12), green plum (*P. cerasifera* Ehr.) (PCR) (0.79 ± 0.28), apricot (*P. armeniaca*) (PAR) (0.81 ± 0.10), and peach (*P. persica*) (PPR) (0.87 ± 0.07) had moderate diuretic activity. Previous studies using *Prunus* species have mostly focused on the fruit extracts of this species. Studies using plant resins are very limited. In their 2024 study, Hema Arya et al. stated that *P. persica* fruit extracts could be used as a potential therapeutic agent for urolithiasis [36]. It has been reported that *P. mahaleb* L. plant resin can be used in the treatment of gastritis [37]. In addition, a study conducted with peach resin reported that the plant resin has diuretic activity [16]. The temporal pharmacological differences identified in our study are considered to stem from characteristic differences in the absorption, distribution, metabolism, and elimination profiles of furosemide and plant resins. The observation of an early diuretic response (5th hour) in the furosemide-treated group is explained by the high absorption rate of the active substance, its minimal metabolic transformation, short terminal half-life, and limited duration of therapeutic effect [38]. In contrast, the increase in urine output in the plant resin group, spread over a later and longer period, is attributed to the slower absorption profile of resin extracts from the gastrointestinal system and the prolonged duration of action (with a long-acting release-like kinetic).

These results point to the potential of plant resin extracts as a supportive alternative to conventional diuretic therapies and suggest that they may contribute to the management of fluid balance in clinical settings. Furthermore, the resin extracts studied, particularly the blackthorn (PSR) resin extract, exhibited somewhat similar trends to furosemide, the reference drug used in our study model, in terms of diuretic effect and diuretic activity capacity.

In the study, the effect of plant resins on urine pH was investigated, and it was found that there were no significant changes in pH levels of treated rats compared to controls [F(7,32) = 1.025, *p* = 0.433] (Table 5). The basal urine pH of control and furosemide-treated rats was measured as 6.50 ± 0.86 and 6.70 ± 0.83, respectively. In rats treated with the plant extract, urine pH was observed to be between 6.92 ± 0.17 and 6.10 ± 0.22, similar to those in the control and furosemide-treated groups. These observations suggest that plant resins may have potential benefits in the management of urinary system dynamics.

The specific gravity of urine (relative density of urine) depends on the amount of dissolved substances, the amount of diuresis, renal tubular function, the amount of fluid ingested, and blood Ca and K concentrations [39]. In the study, urine densities of the treatment groups were measured at the end of 24 h, and significant differences were determined between the groups [F(7,32) = 8.145, *p* = 0.001] (Table 5). The highest density was found in the green plum (PCR) plant resin at 1.36 ± 0.15, and the lowest density was found in the furosemide (F)-treated experimental group. It is thought that the mineral structure of the plant resins is effective in the differences between the groups. It has also been reported that the amount of minerals taken in the daily diet affects urine density [40]. This is because serum potassium and calcium levels are known to affect urine density, with decreases in potassium and increases in serum calcium leading to decreased urine density [41,42]. Furthermore, dehydration, fever, diarrhea, persistent vomiting, proteinuria, glucosuria, certain medications, and some exogenous substances can affect urine density [5,43].

In the study, urine creatinine levels were measured to evaluate the effects of plant resins on kidney function, and significant differences were found between the groups [F(7,32) = 61.681, *p* = 0.001] (Table 5). Urinary creatinine level allows us to understand the kidneys’ capacity to filter blood and remove waste products from the body. A slowdown in the filtering function of the kidneys reduces the creatinine value, while muscle damage and dehydration cause this value to increase. In the current study, total 24 h creatinine excretion was measured only as concentration (mg/dL). The decrease in urinary creatinine concentration (mg/dL) observed in some groups may have been due to a dilution effect resulting from increased urine volume, as well as possible changes in kidney function. In the study, the highest creatinine value was measured in the control group as 183.20 ± 13.08 mg/dL, and the lowest value was measured in the F group treated with furosemide as 79.20 ± 5.54 mg/dL. The creatinine concentration in the urine of a healthy rat generally ranges from 20 to 320 mg/dL on average. Although the values we found were high due to the rats being prevented from accessing water and food during urine collection, they are at a tolerable level. The effects of plant resins on urinary urea levels were investigated, and significant differences were found between the groups [F(7,32) = 28.459, *p* = 0.001] (Table 5). Generally, 24 h urinary urea levels in rats are considered to be between 4000 and 6500 mg/dL. Some of the values we found are lower than these. This is because furosemide and some plant resins administered to rats during the application increased urine volume, causing a decrease in urea concentration. Furthermore, the effects of plant resins on urinary uric acid levels were investigated, and significant differences were found between the groups [F(7,32) = 76.81, *p* = 0.001] (Table 5). Urinary uric acid levels, considered an indicator of purine metabolism and kidney function in the body, are accepted as normal between 2.7 and 5.4 mg/dL on average. The values we found are within the reference values reported in the literature.

Significant differences were found in urine electrolyte content between the groups and are summarized in Table 6 [Na: F(7,32) 17.599, *p* = 0.001; K: F(7,32) 38.506, *p* = 0.001; Cl: F(7,32) 35.793, *p* = 0.001]. The highest urine electrolyte content was calculated in PAR and the lowest in PPR. Interestingly, while the urine electrolyte content calculated in PAR was similar to the control group, the values obtained in PSR and PDuR were closer to the values in the furosemide-treated group. Saluretic activity, characterized by the excretion of sodium, potassium, and chloride ions, exhibited interesting patterns (Table 6). The differences in saluretic activity between groups were significant in terms of the excretion of sodium and chloride ions [Na: F(7,32) 2.335, *p* = 0.048; Cl: F(7,32) 1.847, *p* = 0.032]. However, the differences in the excretion of potassium ions were found to be insignificant [F(7,32) = 1.008, *p* = 0.394]. Although the saluretic effect of plant resins differed between the control and furosemide-treated groups, these differences were tolerable. This demonstrates the diuretic potential of plant resins. Furthermore, these results suggest that the diuretic effect of plant resins is of the saluretic type, unlike the aquaretic type characteristic of most diuretic agents [44]. This can offer advantages in addressing specific clinical scenarios requiring changes in electrolyte balance and sodium excretion.

The natriuretic activity of urine samples collected at 24 h from the study groups was determined, and the differences between the groups were found to be significant [F(7,32) = 2.933, *p* = 0.017] (Table 7). In urine analyses, a Na^+^/K^+^ ratio greater than one indicates satisfactory diuresis without excessive urinary potassium loss. Values greater than 2.0 indicate a positive natriuretic effect; if the ratio exceeds 10.0, it indicates a potassium-sparing effect [45]. All of the values we found were greater than 1 and less than 2. This can be explained as showing satisfactory diuresis without potassium loss in all study groups.

In the study, the ion quotient (Cl^−^/(Na^+^ + K^+^) ratio of the groups was calculated, and the degree of carbonic anhydrase inhibitory effect was shown [F(7,32) = 1.792, *p* = 0.044] (Table 7). Carbonic anhydrase inhibition can be disregarded at ratios between 1.0 and 0.8. As the ratios decrease, carbonic anhydrase inhibition is assumed to be present, from mild to strong [45,46]. Therefore, the fact that the values obtained for plant resins in this study are below 0.8 may provide a preliminary idea or clue about the presence of carbonic anhydrase inhibitory effect and constitute a strong hypothesis for future studies.

## 3. Materials and Methods

The study was carried out in two stages: determining the amount of polyphenols, antioxidant capacity, and mineral content in plant samples, and determining their in vivo diuretic activity.

### 3.1. Collection of Resin Samples and Preparation of Extract

Resin samples were collected in October 2024 from trees in a small family-run fruit farm (40°49′50″ N 42°07′4″ E, 1317 m). The trees from which resin was collected were identified by plant experts working at the facility. After removing physical impurities, the collected samples were dried at room temperature until they reached a constant weight; 5 g of the crushed samples were taken and placed in a sealed Erlenmeyer flask, and 100 mL of methanol was added to the samples and mixed using a magnetic stirrer (WF-M1A, Weightlab Instruments, Istanbul, Türkiye). After filtering the mixture, the solvent was removed at 45 °C using an evaporator (Yamato Scientific Co., Ltd., Tokyo, Japan). Solutions were prepared from the obtained dry crude extract for use in two different analyses. The 1000 ppm stock solution prepared with methanol was used in phytochemical analyses. In determining the in vivo diuretic activity in rats, the dry resin extract dissolved in physiological saline was used [47]. In this study, each resin sample was collected from at least three trees. These samples were then mixed to represent the group and used in the study. Chemical analyses were performed in triplicate for each resin extract. Some of the collected resin voucher specimens are stored as herbarium specimens in the Kırşehir Ahi Evran University Faculty of Health Sciences Laboratory (FE-1001, FE-1002, FE-1003, FE-1004, FE-1005, FE-1006).

### 3.2. Determination of Polyphenol Content

The total phenolic content was determined as gallic acid equivalent using the Folin–Ciocalteu method [48]. The total flavonoid content of the resin samples was determined using the aluminum nitrate method and calculated as quercetin equivalent [49].

### 3.3. Determination of Antioxidant Activity

In this study, 2,6-di-t-butyl-1-hydroxytoluene (BHT) (Merck KGaA, Darmstadt, Germany) was used as a standard for determining antioxidant activity. The DPPH free radical scavenging activity of the resin samples was determined using the Blois (1958) method [50], and IC_50_ values were calculated. In addition, the Fe^3+^-reducing power of the samples was determined [51], and their Cu^2+^-reducing capacities were also measured [52]. To ensure accurate comparability, all resin extracts and the reference antioxidant (BHT) were tested under identical experimental conditions, including incubation time, solvent systems, wavelength, and temperature.

### 3.4. Chromatographic Analyses

Qualitative and quantitative analysis of phenolic compounds in plant resin samples was performed using an HPLC-DAD system (1260 Infinity, Agilent Technologies, Santa Clara, CA, USA) at a detection wavelength of 254 nm. Chromatographic separation was achieved on a Wakosil C18HG column (5 µm, 4.6 × 150 mm, FUJIFILM Wako Pure Chemical Corp., Osaka, Japan) maintained at 40 °C. The mobile phase consisted of water acidified with 0.2% phosphoric acid (Solvent A) and a 50:50 (*v*/*v*) mixture of methanol and acetonitrile (Solvent B), pumped at a flow rate of 1.0 mL/min with an injection volume of 20 µL. The binary gradient elution program was set as follows: 0–40 min, linear gradient from 96% A/4% B to 50% A/50% B; 40–45 min, to 40% A/60% B; 45–60 min, to 0% A/100% B; followed by a 12 min re-equilibration step to initial conditions (96% A/4% B), giving a total run time of 72 min. Phenolic constituents were identified by comparing their retention times and UV spectra with those of reference analytical standards (gallic acid, quercetin, p-oh benzoic acid, chlorogenic acid, vanillic acid, caffeic acid, syringic acid, p-coumaric acid, ferulic acid, rutin, benzoic acid, naringenin, hesperidin, rosmarinic acid and pyrogallol; Sigma-Aldrich, St. Louis, MO, USA) and quantified using the external standard method (mg/kg) [53]. The quantification of target phenolic compounds, particularly hesperidin, was validated in terms of linearity, limit of detection (LOD), limit of quantification (LOQ), and accuracy. External standard calibration curves showed high linearity (R^2^ > 0.999 across the concentration range of 0.5–100 mg/L. The LOD and LOQ values for hesperidin were determined as 0.12 mg/L and 0.38 mg/L (based on S/N = 3 and 10, respectively). Method accuracy was verified through recovery experiments by spiking reference standards into the resin matrix, yielding acceptable recovery values between 95.2% and 103.8%. To maintain the peak areas of high-abundance constituents within the validated linear range, samples were systematically diluted prior to injection.

The identification of volatile and semi-volatile components in plant resin samples was performed using the GC/MS method with a Shimadzu GCMS-QP2010 Ultra instrument (Shimadzu Corp., Kyoto, Japan), Rtx-5MS column (Restek Corp., Bellefonte, PA, USA), helium carrier gas (2 mL/min), oven program increasing from 40 °C to 280 °C, and 1 µL methanol injection parameters in splitless mode [54]. Compound identification was based on spectral matching with mass spectral reference libraries (including NIST11 and Wiley) based on mass spectra similarity, without confirmation using authentic reference standards.

### 3.5. Mineral Analysis (ICP-MS Analysis)

First, 10–20 mg samples were taken and 2 mL HNO_3_ (Merck KGaA, Darmstadt, Germany) and 3 mL H_2_O_2_ (Merck KGaA, Darmstadt, Germany) were added. A temperature and pressure program was applied in the microwave solubilizer (closed system; Milestone Srl, Bergamo, Italy). The clear solutions obtained were taken and diluted to 10 mL with pure water (Milestone Srl, Bergamo, Italy). Before the analysis, standards were prepared at known concentrations (0, 1, 5, 10, 20, 30, 40, 50 ppb) containing the elements to be analyzed. To check the measurement parameters of the device, performance adjustments were made by passing a tune solution (200 ppb Li, Yb, Cs; Agilent Technologies, Santa Clara, CA, USA)) through the device. After the performance of the device was checked with the tune solution, the method containing the elements to be analyzed was selected, and the standards were first introduced to the device, and then the solubilized and diluted samples were analyzed. The entire periodic table was represented during analysis. Apart from the elements to be determined, a solution containing 200 ppb internal standard elements (Sc, In; Agilent Technologies, Santa Clara, CA, USA) was fed to the device. Dissolved samples were analyzed on an Agilent 7900 ICP MS instrument (Agilent Technologies, Santa Clara, CA, USA ) [55].

### 3.6. Determination of the In Vivo Diuretic Properties of Plant Resins

#### 3.6.1. Animal Acquisition and Group Formation

The study was conducted at the Animal Experimentation Unit of Kırşehir Ahi Evran University Faculty of Medicine. The rats used in the study were selected from offspring of the same age group born from unselected breeding stock raised at the Animal Experimentation Unit of Kırşehir Ahi Evran University Faculty of Medicine. They were housed together under standard conditions. Adult Wistar albino rats weighing 200 ± 20 g were used in the study, and the rats were housed under controlled environmental conditions throughout the study: temperature (22 ± 2 °C), relative humidity (65%), and a 12:12 h light/dark cycle (lights on between 08:00 and 20:00).

Before commencing the experiments, the animal use protocol was approved by the Kırşehir Ahi Evran University Local Animal Experiments Ethics Committee (Approval No: 19-03; dated 24 September 2025). All procedures were carried out in accordance with the ARRIVE guidelines (Animal Research: Reporting of In Vivo Experiments). Furthermore, all experimental procedures involving live animals were conducted in accordance with the Regulation on the Welfare and Protection of Animals Used for Experimental and Other Scientific Purposes published by the Ministry of Food, Agriculture, and Forestry of the Republic of Türkiye [56]. In the study, 5 rats treated with the same resin group were considered as one group (Table 8). In this study, the diuretic effects of multiple plant resins were investigated. To validate traditional use and evaluate potential differences in effect, single-dose application was chosen as the most suitable method to improve time and cost efficiency while considering animal welfare [57].

#### 3.6.2. Urine Collection Procedure

In this study, rats in the treatment groups were freely provided with standard rodent feed (Optima, Bolu, Türkiye; 24% crude protein, 3.94% crude cellulose, 5.08% crude fat, 8.8% crude ash, 1.44% lysine, 0.61% methionine, 1.14% calcium, 0.89% phosphorus, 0.28% sodium; Lot No. 58931,) and tap water. To ensure complete emptying of the animals’ bladders and that previous excessive or irregular water consumption did not affect urine measurements, to stabilize initial conditions for accurate measurements, to ensure an empty stomach, to facilitate rapid and even absorption of orally administered resin extracts from the digestive tract, and to eliminate the effect of the feed factor, rats were prevented from accessing water and food for 18 h prior to urine collection. At the end of this period, to ensure a consistent water and salt load in the rats, an oral loading of 2.5 mL/100 g of 0.9% NaCl was administered via gavage (22 gauge; Intech, Shenzhen, China) to all animals [11,58]. Subsequently, rats in the positive control group were orally administered furosemide at a dose of 10 mg/kg, while rats in the treatment group were orally administered an aqueous plant extract at a dose of 100 mg/kg. In this study, the dosage was determined as 100 mg/kg, taking into account the amounts used in previous studies [16]. Following application, rats were placed in individual metabolic cages. Urine samples were collected twice from each rat: at 5 h to represent short-term follow-up and at 24 h to represent long-term follow-up. The experimental protocol was designed to include 5 rats for each resin group and was conducted in a total of 5 replicates.

#### 3.6.3. Determination of Diuretic Effect (DE) and Diuretic Activity (DA)

Urine volumes from rats were collected using metabolic cages (Tecniplast S.p.A., Buguggiate, Italy) and measured using graduated cylinders (ISOLAB Laborgeräte GmbH, Wertheim, Germany). Then, using the urine volumes of the groups, the diuretic effect (DE) and diuretic activity (DA) of the study groups were calculated using the following formulas [61,62].

Diuretic effect = Urine output of the treatment group/Urine output of the control group

Diuretic activity = Urine output of the treatment group/Urine output of the standard drug

#### 3.6.4. Determination of Specific Gravity (SG) and pH

In the study, the urine pH values of the groups were measured at the end of a long period (24 h) using a benchtop pH meter (-MW150 MAX, Milwaukee Instruments, Cluj-Napoca, Romania). Specific gravity values were determined using an optical liquid concentration meter (HI96811, Hanna Instruments, Nușfalău, Romania).

#### 3.6.5. Urine Electrolyte Analysis

Na^+^, K^+^, Cl^−^, urine creatinine, uric acid, and urine urea excreted in the urine of all groups—those treated with plant resin, negative control, and standard medication—were measured using an Ion Selective Electrode (ISE) analyzer (Alinity (AbbottAbbott Park, IL, USA)). Na^+^/K^+^ and Cl^−^/K^+^ + Na^+^ ratios were calculated to assess the natriuretic index and carbonic anhydrase inhibition. The saluretic index was calculated by dividing the urine electrolyte concentration in the group by the urine electrolyte concentration in the control group. Additionally, natriuretic activity and ion quotient values were calculated using the following formulas [35].

$$Natriuretic\;activity=\frac{\left[Na\right]}{\left[K\right]}$$

$$Ion\;quotient=\frac{\left[Cl\right]}{\left[Na+K\right]}$$

### 3.7. Statistical Analyses

The results of the study were analyzed using version 22 of the SPSS software package program. The distribution characteristics of the data were evaluated using the Shapiro–Wilk test for normality and the Levene test for homogeneity of variances. One-way analysis of variance (ANOVA) was applied to data that met the parametric assumptions. In cases where significant differences were detected, the Duncan test, a post hoc test for multiple comparisons, was used to determine the source of the differences between the applications [63]. In addition, a significance level of *p* < 0.05 was used for all calculations in the study.

## 4. Conclusions

In conclusion, our preliminary phytochemical research on plant resins confirmed the presence of valuable metabolites such as phenolic compounds, tannins, flavonoids, cardiac glycosides, and phytosterols and their antioxidant activity. Additionally, the study revealed that plant resins contain potential phytochemicals with significant biological activity. This suggests that plant resins may have rich medicinal value. These findings suggest that plant resins may be responsible for diuretic activity. Furthermore, mineral analysis of the plant resins provided additional information regarding the diuretic potential of the extract. Animals given the plant resin showed no signs of physical illness or death throughout the study. Secondly, the diuretic effect of plant resins was significant; groups treated with plant resin exhibited a high diuretic effect during the treatment period (at 5 h and 24 h). The temporal difference we found in urinary activity is consistent with known properties of diuretic plants, as the duration and activity of diuretic effects produced by plants are generally related to the plant’s components and mechanism of action. In this study, to better understand the mechanism of action of plant resins, they were compared with furosemide, a high-ceiling loop diuretic. It was determined that the resin extracts of blackthorn (*P. spinosa*) (PSR) (1.17 ± 0.09) and almond (*P. dulcis*) (PDuR) (1.00 ± 0.14) had high diuretic activity, while the resin extracts of black plum (*P. domestica*) (PDoR) (0.79 ± 0.12), green plum (*P. cerasifera* Ehr.) (PCR) (0.79 ± 0.28), apricot (*P. armeniaca*) (PAR) (0.81 ± 0.10) and peach (*P. persica*) (PPR) (0.87 ± 0.07) had moderate diuretic activity. Diuresis is characterized by an increase in urine volume and loss of electrolytes excreted in the urine; this is due to the inhibition of the reabsorption of water and electrolytes into the bloodstream in the renal tubules. The furosemide we used as a reference in this study increases urine output and sodium excretion by inhibiting reabsorption in the loop of Henle, the microscopic channels in the kidneys where blood is filtered and converted into urine. The similarity of the results we found regarding the diuretic effects of plant resins to the results in the furosemide-treated group suggests that the possible mechanism of action of plant resins regarding their diuretic effects is similar to that of furosemide. Furthermore, preliminary phytochemical analyses of the plant resins in this study determined the presence of flavonoids, phenols, and antioxidants, and their high levels support this. This is because phenols and flavonoids found in plants are substances that potentially contribute to diuretic activity. This interesting finding highlights the potential of the plant resins used in this study for use in diuretic treatments. This means that they can be used in clinical applications as a strategy for managing fluid balance.

## 5. Limitations of This Study

1. The main limitations of this study are the sample size, the harvest season, the fact that the effects on the bioactive profile were overlooked due to the constancy of extraction processes, and the lack of support for the results by cellular (in vitro) mechanisms.
2. Since this study is an experimental (preliminary) investigation, the sample size was limited to *n* = 5.
3. To minimize the effects of environmental factors on the phytochemical profile, all plant resins examined were collected from the same production area and a narrow geographical region. This allowed for standardization of the potential effects of variables such as soil structure, climate, and harvest season on the results.
4. Multiple plant resins were included in the analysis, and the applications were performed as single doses in accordance with data reported in the literature.
5. Future research plans to purify and isolate bioactive monomers and develop new formulations using these components.
6. The fact that total creatinine and metabolite excretion (mg/24 h) was not calculated is a limitation of this study, and further research examining absolute excretion amounts will provide a clearer explanation of this mechanism.

## Figures and Tables

**Table 1:** Total phenolic and flavonoid content, CUPRAC and FRAP activities, and IC_50_ values.

	TPC (mg GAE/g DE)	TFC (mg QE/g DE)	CUPRAC (mg TE/g)	FRAP (mmol TE/g)	IC_50_ (µg/mL)
PAR	135.00 ± 14.17 ^d^	43.66 ± 7.78 ^b^	462.66 ± 7.57 ^e^	2.15 ± 0.07 ^c^	47.00 ± 2.64 ^c^
PCR	224.00 ± 16.52 ^b^	45.66 ± 1.15 ^b^	649.00 ± 4.88 ^c^	2.70 ± 0.10 ^b^	20.66 ± 2.08 ^e^
PSR	301.33 ± 18.90 ^a^	38.33 ± 3.05 ^c^	660.33 ± 10.51 ^c^	3.61 ± 0.01 ^a^	15.33 ± 2.51 ^f^
PDoR	175.66 ± 9.50 ^c^	32.00 ± 2.64 ^d^	531.33 ± 7.02 ^d^	2.59 ± 0.35 ^b^	36.00 ± 2.00 ^d^
PDuR	208.00 ± 17.57 ^b^	65.00 ± 3.00 ^a^	683.66 ± 18.87 ^b^	3.46 ± 0.05 ^a^	9.66 ± 2.08 ^g^
PPR	37.75 ± 3.78 ^e^	36.66 ± 4.50 ^c^	139.33 ± 14.01 ^f^	0.49 ± 0.02 ^d^	185.66 ± 13.65 ^a^
BHT	-	-	1175.00 ± 56.34 ^a^	3.54 ± 0.04 ^a^	101.66 ± 5.83 ^b^

PAR: *P. armeniaca* resin, PCR: *P. cerasifera Ehr.* resin, PSR: *P. spinosa* resin, PDoR: *P. domestica* resin, PDuR: *P. dulcis* resin, PPR: *P. persica* resin. BHT: 2,6-di-t-butyl-1-hydroxytoluene, TPC: total phenolic activity, GAE: gallic acid equivalent, TFC: total flavonoid activity, QE: quercetin equivalent, CUPRAC: copper (II)-reducing capacity, TE: Trolox equivalent, FRAP: Fe^3+^-Fe^2+^-reducing power, IC_50_: the extract concentration that inhibits 50% of the DPPH radical. Differences between means indicated by the same letter in the same column are statistically insignificant at the *p* < 0.05 level.

**Table 2:** GC/MS analysis table of resin samples.

Samples	Peak	Retention Time	Height%	Name
PAR	1	4.436	0.76	2,3-Butanediol, [R-(R*,R*)]-
	2	9.152	0.63	1,2-Cyclopentanedione
	3	18.350	0.43	Phenol, 2-methoxy- (CAS)
	4	28.006	0.35	2,3-DIHYDRO-BENZOFURAN
	5	33.325	0.44	Phenol, 5-methyl-2-(1-methylethyl)- (CAS)
	6	33.696	0.71	2-Methoxy-4-vinylphenol
	7	36.515	0.49	Phenol, 2,6-dimethoxy-
	8	50.743	0.22	9-Octadecenoic acid (Z)- (CAS)
	9	55.260	0.33	Methyl-(2-hydroxy-3-ethoxy-benzyl)ether
	10	61.942	0.84	n-Hexadecanoic acid
	11	62.272	0.62	Benzoic acid, 4-hydroxy-3,5-dimethoxy-, hydrazide
	12	67.682	2.00	Cycloeicosane
	13	68.934	7.10	Xycaine
	14	70.037	2.13	Hexadecanoic acid, methyl ester (CAS)
	15	70.252	1.45	Benzoic acid, 2-benzoyl-, methyl ester (CAS)
	16	72.130	13.45	2H-1-Benzopyran-2-one, 7-hydroxy-6-methoxy- (CAS)
	17	72.559	11.65	n-Hexadecanoic acid
	18	78.067	1.21	9,12-Octadecadienoic acid, methyl ester
	19	78.369	1.75	9-Octadecenoic acid (Z)-, methyl ester
	20	79.644	1.45	Methyl stearate
	21	80.338	4.07	9,12-Octadecadienoic acid (Z,Z)-
	22	80.635	7.86	cis-Vaccenic acid
	23	81.744	7.28	9-Octadecenoic acid (Z)- (CAS)
	24	83.463	0.81	Methyl 16-hydroxy-hexadecanoate
	25	86.677	2.63	Nonacosanol (CAS)
	26	90.246	2.32	Eicosanoic acid
	27	90.900	0.49	1-Eicosanol
	28	93.243	2.87	Dehydroabietic acid
	29	95.055	6.09	1-Heneicosanol
	30	96.601	1.92	Octadecanoic acid, methyl ester (CAS)
	31	98.257	1.76	Docosanoic acid (CAS)
	32	101.431	3.76	Stigmast-5-en-3-ol, (3.beta.)- (CAS)
	33	101.699	3.90	.beta.-Sitosterol
	34	114.921	0.59	Lanosterol
	35	115.325	2.85	Cholesta-4,6-dien-3-ol, (3.beta.)-
	36	116.023	1.09	Cholest-5-en-3-ol (3.beta.), carbonochloridate
	37	116.802	1.66	1,1,6-trimethyl-3-methylene-2-(3,6,9,13-tetramethyl
PCR	1	2.720	2.07	Cyclohexanol, 3-(3,3-dimethylbutyl)- (CAS)
	2	3.199	2.90	Acetic acid, hydroxy-, methyl ester (CAS)
	3	3.523	7.99	1,2,3-Propanetriol (CAS)
	4	3.649	5.44	Propanoic acid, 2-hydroxy-, methyl ester, (+/−)-
	5	3.883	10.50	Pyrrole
	6	4.090	1.33	Pyridine (CAS)
	7	11.804	1.46	Carbamic acid, methyl-, phenyl ester
	8	18.612	1.35	Benzoic acid, methyl ester (CAS)
	9	36.500	1.41	Phenol, 2,6-dimethoxy-
	10	70.012	9.55	Hexadecanoic acid, methyl ester
	11	70.207	0.94	Benzoic acid, 2-benzoyl-, methyl ester (CAS)
	12	72.071	8.33	n-Hexadecanoic acid
	13	77.541	0.96	1-Hexadecanol (CAS)
	14	78.007	1.30	9,12-Octadecadienoic acid, methyl ester
	15	78.321	4.18	9-Octadecenoic acid, methyl ester, (E)- (CAS)
	16	79.595	5.21	Methyl stearate
	17	81.377	1.38	9-Octadecenoic acid (Z)- (CAS)
	18	82.912	1.32	Retene
	19	105.847	32.38	9-Octadecenamide, (Z)-
PSR	1	2.603	9.77	Acetic acid
	2	3.222	5.33	Acetic acid, hydroxy-, methyl ester
	3	3.553	10.85	Glycerin
	4	3.675	3.56	2-Propanol (CAS)
	5	3.862	3.51	2-Propenoic acid, methyl ester (CAS)
	6	5.595	2.28	Furfural
	7	27.970	1.50	2,3-DIHYDRO-BENZOFURAN
	8	63.852	1.12	Ethanone, 1-(2,6-dihydroxy-4-methoxyphenyl)- (CAS)
	9	70.020	3.90	Hexadecanoic acid, methyl ester
	10	72.256	7.89	n-Hexadecanoic acid
	11	77.567	1.56	1-Octadecanol
	12	79.624	2.68	Methyl stearate
	13	80.320	1.84	9-Octadecenoic acid (Z)- (CAS)
	14	81.501	3.70	9-Octadecenoic acid (Z)- (CAS)
	15	86.654	0.92	1-Heptacosanol (CAS)
	16	95.012	4.90	1-Heneicosanol
	17	95.797	0.87	Hexadecanoic acid, 2-hydroxy-1-(hydroxymethyl)ethyl ester
	18	102.756	3.08	1-Heneicosanol
	19	103.635	2.46	Octadecanoic acid, 2,3-dihydroxypropyl ester
	20	104.114	0.69	Triacontanoic acid, methyl ester
	21	105.943	24.02	9-Octadecenamide, (Z)-
	22	109.962	2.26	1-Octacosanol (CAS)
	23	115.979	1.31	Cholest-5-ene, 3-bromo-, (3.beta.)-
PDoR	1	2.595	4.05	Acetic acid (CAS)
	2	3.583	7.43	Glycerin
	3	3.711	2.21	PROPANOIC ACID, 2-HYDROXY-, METHYL ESTER
	4	33.310	0.54	Phenol, 2-methyl-5-(1-methylethyl)- (CAS)
	5	33.691	0.55	2-Methoxy-4-vinylphenol
	6	41.802	0.96	Coumarin
	7	65.839	0.84	2-Pentadecanone, 6,10,14-trimethyl-
	8	67.671	1.31	1-Hexadecanol
	9	68.916	8.53	Xycaine
	10	70.030	4.53	Hexadecanoic acid, methyl ester
	11	70.249	1.62	Benzoic acid, 2-benzoyl-, methyl ester
	12	72.454	13.45	n-Hexadecanoic acid
	13	77.586	1.87	1-Octadecanol (CAS)
	14	78.058	1.46	11,14-Eicosadienoic acid, methyl ester
	15	78.358	2.72	9-Octadecenoic acid, methyl ester, (E)- (CAS)
	16	79.637	3.08	Methyl stearate
	17	80.148	2.33	9,12-Octadecadienoic acid (Z,Z)-
	18	83.442	1.22	Methyl 16-hydroxy-hexadecanoate
	19	85.416	1.02	Docosanedioic acid, dimethyl ester
	20	90.179	0.84	9-Octadecenoic acid (Z)- (CAS)
	21	95.050	8.82	1-Heneicosanol
	22	101.353	7.07	Stigmast-5-en-3-ol, (3.beta.)- (CAS)
	23	102.783	3.11	Octacosanol
	24	103.708	7.70	Octadecanoic acid, 2,3-dihydroxypropyl ester
	25	109.991	2.04	1-Octacosanol (CAS)
	26	114.276	5.56	Stigmast-4-en-3-one
	27	115.326	5.14	Cholesta-4,6-dien-3-ol, (3.beta.)-
PDuR	1	2.636	5.90	Acetic acid (CAS)
	2	3.267	1.96	Acetic acid, hydroxy-, methyl ester (CAS)
	3	3.603	6.99	1,2,3-Propanetriol (CAS)
	4	3.731	2.19	Propanoic acid, 2-hydroxy-, methyl ester, (+/−)-
	5	9.171	3.28	1,2-Cyclopentanedione
	6	19.329	1.02	ADAMANTOL-(1)
	7	27.445	0.77	1,2-Benzenediol (CAS)
	8	27.522	0.59	Catechol
	9	27.975	0.64	2,3-DIHYDRO-BENZOFURAN
	10	61.863	0.87	Tridecanoic acid
	11	67.670	0.86	Cycloeicosane
	12	70.029	3.07	Hexadecanoic acid, methyl ester (CAS)
	13	72.308	10.44	n-Hexadecanoic acid
	14	78.028	0.64	9,12-Octadecadienoic acid, methyl ester
	15	78.330	1.85	9-Octadecenoic acid, methyl ester, (E)- (CAS)
	16	79.618	1.66	Methyl stearate
	17	80.400	5.14	9-Octadecenoic acid (Z)- (CAS)
	18	80.591	1.38	cis-Vaccenic acid
	19	86.663	0.75	7-Hexadecanoic acid, methyl ester, (Z)-
	20	95.764	0.88	Hexadecanoic acid, 2-hydroxy-1-(hydroxymethyl)ethyl ester
	21	101.309	5.56	.beta.-Sitosterol
	22	102.751	1.18	1-Heneicosanol
	23	105.951	30.97	9-Octadecenamide, (Z)-
	24	109.545	2.96	Naringenin
	25	109.976	4.08	Octacosanol
	26	115.301	2.21	Cholesta-4,6-dien-3-ol, (3.beta.)-
PPR	1	2.698	3.87	Acetic acid
	2	2.965	1.08	1-propylmethyl ether
	3	3.274	1.56	1,2-Butanediol
	4	3.612	3.88	Glycerin
	5	3.905	1.59	2-Propenoic acid, methyl ester (CAS)
	6	9.274	1.58	1,2-Cyclopentanedione
	7	12.533	2.02	PHENOL
	8	67.821	19.89	Cycloeicosane
	9	72.831	8.00	n-Hexadecanoic acid
	10	80.205	0.69	9,12-Octadecadienoic acid (Z,Z)-
	11	80.604	3.36	cis-Vaccenic acid
	12	80.825	1.28	cis-9-Hexadecenoic acid
	13	81.842	4.97	Octadecanoic acid
	14	87.750	0.73	E-11-Hexadecenal
	15	88.450	0.46	Eicosanoic acid, methyl ester (CAS)
	16	88.771	0.54	Cyclohexane, eicosyl-
	17	90.324	2.75	Eicosanoic acid
	18	94.251	0.71	9-Octadecenoic acid (Z)- (CAS)
	19	98.339	2.77	Docosanoic acid (CAS)
	20	101.052	0.83	.beta.-Sitosterol
	21	106.110	10.93	9-Octadecenamide, (Z)-
	22	109.448	1.15	(R)-(-)-14-Methyl-8-hexadecyn-1-ol
	23	110.240	16.56	Octacosanol
	24	113.407	4.23	Hexadecanoic acid, ethyl ester (CAS)
	25	115.389	4.09	Cholesta-4,6-dien-3-ol, (3.beta.)-
	26	116.045	0.48	Cholest-5-ene, 3-bromo-, (3.beta.)-

**Table 3:** ICP-MS analysis table (ppm).

	Na	Mg	P	K	Ca	Cr	Mn	Fe	Cu	Zn
PAR	167.85	2050.69	1473.39	2010.18	4619.15	1.60	4.84	102.10	3.33	3.95
PCR	165.67	2076.37	1417.72	4901.81	2206.00	1.13	30.11	67.55	1.88	0.29
PSR	81.10	1669.91	715.53	2104.33	3648.00	0.83	21.37	424.57	4.32	1.44
PDoR	112.84	1751.52	1064.02	3015.90	3423.02	1.20	16.59	104.09	1.94	17.77
PDuR	124.91	2028.75	1009.71	2145.02	3856.22	1.08	31.73	134.50	1.02	2.74
PPR	126.32	1078.16	1114.73	1557.32	1623.19	1.42	36.42	198.59	2.26	8.63

PAR: *P. armeniaca* resin, PCR: *P. cerasifera Ehr.* resin, PSR: *P. spinosa* resin, PDoR: *P. domestica* resin, PDuR: *P. dulcis* resin, PPR: *P. persica* resin, Na: Sodium, Mg: Magnesium, P: Phosphorus, K: Potassium, Ca: Calcium, Cr: Chromium, Mn: Manganese, Fe: Iron, Cu: Copper, Zn: Zinc.

**Table 4:** Urine volume, diuretic effect and diuretic activity.

	5 h			24 h		
Groups	V (mL/100 g/5 h)	DE	DA	V (mL/100 g/24 h)	DE	DA
C	1.21 ± 0.09 ^c^	1 ^c^	0.48 ± 0.06 ^c^	1.65 ± 0.10 ^e^	1 ^e^	0.50 ± 0.05 ^d^
F	2.49 ± 0.24 ^a^	2.06 ± 0.11 ^a^	1 ^a^	3.30 ± 0.27 ^ab^	1.99 ± 0.21 ^ab^	1 ^ab^
PAR	1.09 ± 0.10 ^c^	0.90 ± 0.19 ^c^	0.44 ± 0.07 ^c^	2.70 ± 0.48 ^bcd^	1.64 ± 0.34 ^bcd^	0.81 ± 0.10 ^c^
PCR	1.20 ± 0.16 ^c^	0.99 ± 0,32 ^c^	0.48 ± 0.10 ^c^	2.61 ± 076 ^cd^	1.57 ± 0.43 ^cd^	0.79 ± 0.28 ^c^
PSR	1.88 ± 0.21 ^b^	1.55 ± 0.26 ^b^	0.76 ± 0.14 ^b^	3.64 ± 0.55 ^a^	2.29 ± 0.23 ^a^	1.17 ± 0.09 ^a^
PDoR	1.19 ± 0.23 ^c^	0.79 ± 0.19 ^c^	0.50 ± 0.11 ^c^	2.37 ± 0.32 ^d^	1.43 ± 0.22 ^d^	0.79 ± 0.12 ^c^
PDuR	1.10 ± 0.18 ^c^	0.90 ± 0,15 ^c^	0.43 ± 0.09 ^c^	3.09 ± 0.26 ^abc^	1.87 ± 0.26 ^bc^	1.00 ± 0.14 ^ab^
PPR	0.96 ± 0.21 ^c^	0.78 ± 0.22 ^c^	0.38 ± 0.07 ^c^	2.91 ± 0.30 ^bcd^	1.76 ± 0.22 ^bcd^	0.87 ± 0.07 ^bc^

C: control, F: furosemide, PAR: *P. armeniaca* resin, PCR: *P. cerasifera Ehr.* resin, PSR: *P. spinosa* resin, PDoR: *P. domestica* resin, PDuR: *P. dulcis* resin, PPR: *P. persica* resin, V: volume, DE: diuretic effect, DA: diuretic activity. Differences between means indicated by the same letter in the same column are statistically insignificant at the *p* < 0.05 level.

**Table 5:** Urine pH, specific gravity, urine creatinine, uric acid and urine urea levels.

Groups	pH	SG	Urine Creatinine (mg/dL)	Uric Acid (mg/dL)	Urine Urea (mg/dL)
C	6.67 ± 2.29 ^ab^	1.34 ± 0.19 ^a^	183.20 ± 13.08 ^a^	3.52 ± 1.78 ^bc^	4806.70 ± 146.72 ^b^
F	6.89 ± 2.12 ^ab^	0.92 ± 0.06 ^cd^	79.20 ± 5.54 ^e^	5.32 ± 0.16 ^a^	3848.40 ± 96.53 ^c^
PAR	6.27 ± 1.97 ^ab^	1.09 ± 0.07 ^bc^	104.80 ± 12.85 ^c^	5.20 ± 1.70 ^a^	3950.70 ± 54.77 ^c^
PCR	6.36 ± 0.30 ^ab^	1.36 ± 0.15 ^a^	127.20 ± 5.58 ^b^	4.18 ± 1.36 ^ab^	6987.00 ± 171.28 ^a^
PSR	7.32 ± 1.14 ^a^	0.87 ± 0.08 ^d^	97.20 ± 8.58 ^cd^	2.30 ± 0.76 ^c^	3558.90 ± 508.83 ^c^
PDoR	7.68 ± 1.42 ^a^	1.04 ± 0.06 ^cd^	118.00 ± 12.02 ^b^	2.36 ± 0.36 ^c^	3970.00 ± 54.77 ^c^
PDuR	6.25 ± 0.81 ^ab^	1.26 ± 0.01 ^ab^	89.40 ± 5.50 ^de^	2.30 ± 0.36 ^c^	4146.00 ± 177.98 ^c^
PPR	5.42 ± 087 ^b^	0.95 ± 0.02 ^cd^	99.00 ± 5.95 ^cd^	2.20 ± 0.25 ^c^	4020.00 ± 19.78 ^c^

C: control, *F*: furosemide, PAR: *P. armeniaca* resin, PCR: *P. cerasifera Ehr.* resin, PSR: *P. spinosa* resin, PDoR: *P. domestica* resin, PDuR: *P. dulcis* resin, PPR: *P. persica* resin, SG: specific gravity; differences between means indicated by the same letter in the same column are statistically insignificant at the *p* < 0.05 level.

**Table 6:** Urinary electrolyte content and saluretic index.

	Urinary Electrolyte Excretion			Saluretic Index		
	Na^+^	K^+^	Cl^−^	Na^+^	K^+^	Cl^−^
C	198.40 ± 7.73 ^ab^	153.00 ± 6.44 ^a^	240.60 ± 8.79 ^b^	1 ^abc^	1 ^a^	1 ^ab^
F	153.40 ± 12.09 ^c^	127.80 ± 2.48 ^c^	176.20 ± 4.14 ^cd^	0.79 ± 0.18 ^bc^	0.92 ± 0.31 ^a^	0.89 ± 0.22 ^ab^
PAR	208.20 ± 21.34 ^a^	150.20 ± 5.44 ^a^	234.00 ± 16.67 ^a^	1.06 ± 0.26 ^ab^	1.09 ± 0.43 ^a^	1.03 ± 0.27 ^a^
PCR	182.90 ± 12.39 ^b^	138.60 ± 5.129 ^b^	203.90 ± 10.59 ^b^	0.97 ± 0.27 ^abc^	1.04 ± 0.33 ^a^	1.00 ± 0.35 ^ab^
PSR	151.00 ± 8.45 ^c^	114.60 ± 6.50 ^d^	164.80 ± 12.87 ^d^	0.76 ± 0.16 ^bc^	0.81 ± 0.25 ^a^	0.79 ± 0.08 ^ab^
PDoR	207.80 ± 21.43 ^a^	134.80 ± 7.89 ^bc^	200.80 ± 7.19 ^b^	1.14 ± 0.39 ^a^	0.96 ± 0.30 ^a^	1.05 ± 0.09 ^a^
PDuR	158.40 ± 12.44 ^c^	111.60 ± 7.16 ^d^	187.60 ± 6.06 ^c^	0.83 ± 0.17 ^abc^	0.78 ± 0.15 ^a^	0.92 ± 0.04 ^ab^
PPR	143.10 ± 6.94 ^c^	107.00 ± 7.48 ^d^	149.80 ± 6.41 ^c^	0.72 ± 0.10 ^d^	0.75 ± 0.06 ^a^	0.74 ± 0.10 ^b^

C: control, F: furosemide, PAR: *P. armeniaca* resin, PCR: *P.cerasifera Ehr.* resin, PSR: *P. spinosa* resin, PDoR: *P. domestica* resin, PDuR: *P. dulcis* resin, PPR: *P. persica* resin. Differences between means indicated by the same letter in the same column are statistically insignificant at the *p* < 0.05 level.

**Table 7:** Na^+^ + Cl^−^, natriuretic activity and ion quotient table.

		Natriuretic Activity	Ion Quotient
	Na^+^ + Cl^−^	Na^+^/K^+^	Cl/(Na^+^ + K^+^)
C	401.05 ± 69.66 ^abc^	1.37 ± 0.19 ^abc^	1 ^a^
F	335.60 ± 18.52 ^bcd^	1.21 ± 0.15 ^c^	0.87 ± 0.33 ^a^
PAR	418.40 ± 96.50 ^ab^	1.25 ± 0.04 ^c^	0.80 ± 0.04 ^a^
PCR	381.00 ± 69.40 ^abc^	1.38 ± 0.10 ^bc^	0.19 ± 0.08 ^b^
PSR	314.00 ± 70.26 ^cd^	1.30 ± 0.19 ^bc^	0.34 ± 0.01 ^b^
PDoR	444.80 ± 72.06 ^a^	1.63 ± 0.33 ^a^	0.26 ± 0.09 ^b^
PDuR	353.80 ± 66.61 ^abcd^	1.55 ± 0.15 ^ab^	0.22 ± 0.04 ^b^
PPR	293.40 ± 37.71 ^d^	1.38 ± 0.19 ^abc^	0.47 ± 0.05 ^b^

C: control, F: furosemide, PAR: *P. armeniaca -* resin, PCR: *P. cerasifera Ehr.* resin, PSR: *P. spinosa* resin, PDoR: *P. domestica* resin, PDuR: *P. dulcis* resin, PPR: *P. persica* resin. Differences between means indicated by the same letter in the same column are statistically insignificant at the *p* < 0.05 level.

**Table 8:** Working groups.

Groups	Procedure
C	-%0.9 NaCl (2.5 mL/100 g oral) [58,59]
F	-%0.9 NaCl (2.5 mL/100 g oral) + Furosemide (10 mg/kg orally) [60]
PAR	-%0.9 NaCl (2.5 mL/100 g oral) + Resin Extract (100 mg/kg orally)
PCR	-%0.9 NaCl (2.5 mL/100 g oral) + Resin Extract (100 mg/kg orally)
PSR	-%0.9 NaCl (2.5 mL/100 g oral) + Resin Extract (100 mg/kg orally)
PDoR	-%0.9 NaCl (2.5 mL/100 g oral) + Resin Extract (100 mg/kg orally)
PDuR	-%0.9 NaCl (2.5 mL/100 g oral) + Resin Extract (100 mg/kg orally)
PPR	-%0.9 NaCl (2.5 mL/100 g oral) + Resin Extract (100 mg/kg orally)

C: control, F: furosemide, PAR: *P. armeniaca* resin, PCR: *P. cerasifera Ehr.* resin, PSR: *P. spinosa resin*, PDoR: *P. domestica* resin, PDuR: *P. dulcis* resin, PPR: *P. persica* resin.

## Data Availability

All data generated or analyzed during this study are included in this published article.

## Supplementary Materials

The following supporting information can be downloaded at https://www.mdpi.com/article/10.3390/molecules31173004/s1. Table S1. Retention times and concentrations of individual phenolic compounds identified in *Prunus* resins by HPLC-DAD.
