# Supplementary material for: Determination of Chemical Composition and Antioxidant Capacity of Fruit Tree Resins Belonging to the Prunus Species and Determination of Their In Vivo Diuretic Activities
Source: Molecules. 2026 Aug 27;31(17):3004. doi: 10.3390/molecules31173004 (PMC13567464; doi:10.3390/molecules31173004)
Supplement: Supplementary file 1 [file molecules-31-03004-s001.zip › molecules-4504180-supplementary.pdf]

Supplementary Table S1. Retention times and concentrations of individual phenolic compounds identified in Prunus resins by HPLC-DAD

Phenolic	PAR		PCR		PSR		PDoR		PDuR		PPR	
**Compound**	**RT**	**Conc.**	**RT**	**Conc.**	**RT**	**Conc.**	**RT**	**Conc.**	**RT**	**Conc.**	**RT**	**Conc.**
Gallic acid	6.701	nd	6.701	nd	6.701	nd	6.701	nd	6.701	nd	6.705	99.89
Quercetin	10.888	231.69	10.725	106.30	10.825	117.76	10.878	151.52	10.853	171,94	10.705	396.20
p-OH benzoic acid	15.022	7.09	14.813	4.19	14.942	6.26	14.998	7.63	14.990	4.42	14.768	46.31
Chlorogenic acid	16.379	33.59	16.239	49.98	16.538	65.66	15.940	850.29	15.836	701.88	16.174	250.18
Vanillic acid	17.107	203.74	17.204	2.69	17.031	23.31	17.085	17.22	17.065	28.98	17.193	174.91
Caffeic acid	18.000	nd	18.149	9.25	18.00	nd	18.00	nd	17.883	122.86	17.692	348.36
Syringic acid	18.319	66.16	18.45	15.61	18.191	8.69	18.282	4.78	18.302	nd	18.179	103.26
p-Coumaric acid	22.185	1449.23	21.944	4686.48	22.082	2046.90	22.196	4707.44	22.131	2741.02	22.403	1284.43
Ferulic acid	23.121	3636.38	22.906	61.68	23.037	987.60	22.951	460.57	22.751	nd	22.925	79.99
Rutin	24.477	481.42	24.232	1081.91	24.402	465.34	24.457	399.70	24.435	115.48	23.864	56.25
Benzoic acid	25.975	239.01	26.463	14.72	26.55	81.29	25.975	300.68	26.589	nd	26.581	308.44
Naringenin	26.928	19.02	27.068	384.38	27.288	898.41	27.313	808.85	26.738	132.41	27.110	1071.99
Hesperidin	29.030	10166.60	28.433	1729.53	29.063	2171.79	29.049	769.48	28.938	30177.1	28.606	35289.8
Rosmarinic acid	30.551	3161.65	30.902	33.21	30.480	18.86	30.323	57.65	30.554	236.64	30.547	286.33
Pyrogallol	35.090	1.71	35.811	nd	34.824	26.53	34.947	38.95	35.881	nd	34.600	66.54

PAR; *P. armeniaca* resin, PCR; *P. cerasifera Ehr.* resin, PSR; *P. spinosa* resin, PDoR; *P. domestica* resin, PDuR; *P. dulcis* resin, PPR; *P. persica* resin, RT; Retention Time (min), Conc.; Concentration (mg/kg dry weight)
